# miR-632 promotes gastric cancer progression by accelerating angiogenesis in a TFF1-dependent manner

**DOI:** 10.1186/s12885-018-5247-z

**Published:** 2019-01-07

**Authors:** Ying Shi, Xiaoxiao Huang, Guobin Chen, Ying Wang, Yuansheng Liu, Wei Xu, Shaohui Tang, Bayasi Guleng, Jingjing Liu, Jianlin Ren

**Affiliations:** 1 Department of Gastroenterology, The First Affiliated Hospital, Jinan University, Guangzhou, 510630 People’s Republic of China; 2 The First Clinical Medical College, Jinan University, Guangzhou, 510630 People’s Republic of China; 3 Department of Gastroenterology, Zhongshan Hospital, Xiamen University, Xiamen, 361004 People’s Republic of China; 4 Xiamen branch, Zhongshan hospital, Fudan University, Xiamen, 361015 People’s Republic of China; 5 Department of Gastroenterology, The Affiliated Hospital of Guizhou Medical University, Guiyang, 550004 People’s Republic of China

**Keywords:** miR-632, Trefoil factor 1, Gastric cancer, Angiogenesis

## Abstract

**Background:**

Gastric cancer (GC) is a common malignant disease worldwide. Aberrant miRNAs expression contributes to malignant cells behaviour, and in preclinical research, miRNA targeting has shown potential for improving GC therapy. Our present study demonstrated that miR-632 promotes GC progression in a trefoil factor 1 (TFF1)-dependent manner.

**Methods:**

We collected GC tissues and serum samples to detect miR-632 expression using real-time PCR. A dual-luciferase reporter assay was used to identify whether miR-632 directly regulates TFF1 expression. Tube formation and endothelial cell recruitment assays were performed with or without miR-632 treatment. Western blot and in situ hybridization assays were performed to detect angiogenesis and endothelial recruitment markers that are affected by miR-632.

**Results:**

Our results showed that miR-632 is highly expressed in GC tissue and serum and negatively associated with TFF1 in GC. miR-632 improves tube formation and endothelial cell recruitment by negatively regulating TFF1 in GC cells. Recombinant TFF1 reversed miR-632-mediated angiogenesis. TFF1 is a target gene of miR-632.

**Conclusions:**

Our study demonstrated that miR-632 promotes GC progression by accelerating angiogenesis in a TFF1-dependent manner. Targeting of miR-632 may be a potential therapeutic approach for GC patients.

**Electronic supplementary material:**

The online version of this article (10.1186/s12885-018-5247-z) contains supplementary material, which is available to authorized users.

## Background

Gastric cancer (GC) is a common malignant disease worldwide [1, 2]. Cancer cells invade locally and metastasize to distant sites, which leads to death [3]. A better understanding of factors that contribute to GC cell behaviour could potentially improve GC therapies. Two decades ago, microRNA (miRNA) was discovered to be a non-coding RNA and identified as a regulatory gene [4, 5]. Currently, miRNAs are reported to regulate the development of many diseases, especially malignant tumours, by acting as either oncogenes or tumour suppressors [6–8]. miRNA dysregulation is involved in cancer progression and may provide targets for novel therapeutic approaches [9–12]. Several miRNA-targeted therapeutic strategies have reached clinical or preclinical development [13]. Currently, a miRNA-associated signature provides predictive power to classify and stratify EGC patients for endoscopic treatment [14]. A mimic of the tumour suppressor miR-34 has reached phase I clinical trials for cancer treatment. Anti-miR-122 therapy has reached phase II trials for hepatitis treatment [15]. Silencing of miR-632 inhibits EMT (epithelial-mesenchymal transition) and eliminates invasive ability in breast cancer cells. In addition, miR-632 is related to nasopharyngeal carcinoma, laryngeal cancer and myelodysplastic syndrome [16–18]. Tuberculosis risk may be influenced by miR-632-mediated regulation [19]. However, the relationship between miR-632 and GC therapeutics still needs to be clarified.

The trefoil factor family is a group of small-molecule polypeptides secreted by the mammalian gastrointestinal tract [20]. Trefoil factor 1 (TFF1), a member of the trefoil peptide family, has been reported to inhibit gastrointestinal tumourigenesis. TFF1 is highly expressed in the human stomach and maintains gastric epithelial structure and function [21]. However, this tissue-specific distribution is disrupted in pathological states. In metastatic GC, TFF1 is upregulated compared with its expression in primary cancer [22]. After GC resection, secreted TFF1 in serum acts as a recurrence biomarker [23]. We previously explored the effect of TFF1 in the maintenance of gastric mucosa integrity and continuity and found that TFF1 is closely associated with GC progression [24, 25]. According to a computer-based set of predictions, target sequences within the TFF gene cluster demonstrate that multiple miRNAs can potentially bind the 3′-untranslated region and DNA coding sequence [26]. We previously showed that miR-423-5p and miR-218-5p regulate GC proliferation and invasion by targeting TFF1, respectively [27, 28].

Aberrant miRNA expression contributes to malignant cell behaviour, and in preclinical research, miRNA targeting has shown potential for improving GC therapy. Here, we demonstrate that miR-632 promotes tumour angiogenesis and endothelial recruitment in a TFF1-dependent manner.

## Methods

### Ethics statement

This study was approved by the Ethics Committee of The First Affiliated Hospital, Jinan University, China. Written consent was obtained from all participants.

### Cell culture and transfection

AGS cell lines were purchased from ATCC (Manassas, VA, USA) and cultured in Ham’s F-12 K (Kaighn’s) medium (Life Technologies) supplemented with 10% foetal bovine serum (FBS, Life Technologies) and 1% penicillin G/streptomycin (Life Technologies). BGC823, MGC803, MKN45 and EAhy926 cell lines were purchased from Cell Bank, Shanghai Institutes for Biological Sciences (Cell Bank, CAS, Shanghai, China) and cultured in RPMI 1640 medium (Life Technologies) supplemented with 10% FBS and 1% penicillin G/streptomycin. All cell lines were incubated at 37 °C in an atmosphere of 95% air and 5% CO_2_. The cell lines were checked free of mycoplasma contamination by PCR and culture, and authenticated with STR profiling (FBI, CODIS, http://cellresource.cn).

miR-632-mimic (25 nM) and miR-632-inhibitor (50 nM) were purchased from Qiagen. The cell transfection method was described previously [28].

### RNA expression analysis

A miRNeasy Kit (Qiagen; cat. no. 217004) was used for total RNA extraction from human GC cells or tissues, and a miRNeasy Serum/Plasma Kit (Qiagen; cat. no. 217184) was used for miRNA extraction from GC patient serum following the manufacturer’s protocol. miRNA first-strand cDNA synthesis and real-time PCR were performed as previously described [28]. miR-632 and control primers were purchased from Qiagen. TFF1 primers were described previously [27].

### In situ hybridization and immunohistochemical staining

Digoxin-labelled hsa-miR-632 probe (miRCURY LNA™ Detection probe; 250 pmol; 5’-DIG and 3’-DIG labelled; Exiqon) was used at a concentration of 1.5 pM to detect miR-632 expression. Rabbit anti-human TFF1 polyclonal antibody (ab92377) was purchased from Abcam and diluted 1:250 in IHC Antibody Diluent (ABD-0030; Maixin Biotech, Fuzhou, China). Anti-MMP9 (MAB-0245) and anti-CD34 (MAB-0034) antibodies were purchased from Maixin Biotech, Fuzhou, China. In situ hybridization and immunohistochemical staining were performed in serial paraffin sections of human GC tissue using previously described procedures [28].

### Western blot analysis

GC cells were transfected with miR-632 mimic or inhibitor or with corresponding controls for 48 h. Then, the cells were collected and lysed for Western blot analysis. Primary antibodies against TFF1 were purchased from Santa Cruz Biotechnology, and antibodies targeting MMP9, CD34, p-NFκB, and NFκB were purchased from Cell Signalling Technology. Primary antibodies against GAPDH and anti-rabbit/mouse secondary antibodies were purchased from Proteintech.

### Dual-luciferase reporter assay

AGS cells were seeded in 24-well plates at a density of 6 × 10^4^ cells per well immediately prior to transfection. pmirGLO dual-luciferase miRNA target expression vector was purchased from Promega (cat. E1330). The full-length or a mutated 3’UTR region of TFF1 was inserted into a luciferase reporter vector. AGS cells were co-transfected with miR-632-mimic along with the vectors. After 48 h, the cells were assessed for both firefly and Renilla luciferase activity using a dual-luciferase reporter assay system (Promega; E1910).

### Enzyme-linked immunosorbent assay (ELISA)

After 24 h of miR-632 mimic or inhibitor treatment, cell supernatants were collected and examined using a TFF1 ELISA kit (USCN Life Science, Houston, TX, USA) following the manufacturer’s protocol. The absorbance at 450 nm was measured using a microplate reader.

### Endothelial recruitment experiment

EAHY926 cells were grown to 100% confluency in 60-mm dishes. Three coverslips seeded with GC cells transfected with hsa-miR-632 mimic, hsa-miR-632 inhibitor or corresponding controls were transferred onto the EAHY926 monolayer and a scratch was made across the EAHY926 monolayer using a 200-μl pipet tip. At least three images were collected along each scratch and analysed for the area covered by EAHY926 cells.

### Blood tube formation assays

Gastric cells were treated with hsa-miR-632-mimic, hsa-miR-632-inhibitor or corresponding controls and incubated at 37 °C for 24 h. The supernatant was collected and added to EAHY926 cells. A total of 100 μl Matrigel (BD Bioscience) was added to a 96-well plate and allowed to polymerize. Then, 3 × 10^4^ EAHY926 cells in serum-free medium were added to each Matrigel-coated well. Cells were incubated for 6 h at 37 °C and then imaged via microscopy. Each group was evaluated in triplicate.

### Cell migration and wound healing assays

Cell migration was analysed using Transwell and wound healing assays. The Transwell assay was performed using 24-well Transwell plates (Costar, USA) polycarbonate filters containing 8-μm-poros. Briefly, 200 μl of cell suspension containing 1 × 10^5^ cells in the absence of FBS was added into each upper Transwell chamber, and 500 μl of medium containing 10% FBS was added to the lower chamber. The cells were incubated for 20 h and then fixed in 4% paraformaldehyde for 10 min. Cells were stained with 1% crystal violet for 30 min and counted.

Wound healing assays were performed in 6-well plates. Cells were cultured in 6-well plates at a density of 5 × 10^6^ cells per well for 24 h, and then, a scratch was made across the cell monolayer with a 200-μl pipet tip. At least three images were collected along each scratch and analysed for the area covered by cells.

### Statistical analysis

Statistical analysis was performed using SPSS 21.0 software (SPSS Inc., Chicago, IL, USA). Student’s t-test (means ± standard deviation) and chi-square test were used for data analysis according to different data types. All of the values are expressed as the mean ± SD of at least three independent experiments performed in triplicate, and *P* < 0.05 was considered to be statistically significant. Graphs were plotted using GraphPad Prism 5.0 software (GraphPad Software Inc., La Jolla, CA, USA).

## Results

### miR-632 is highly expressed in GC tissue and serum

**Fig. 1:**
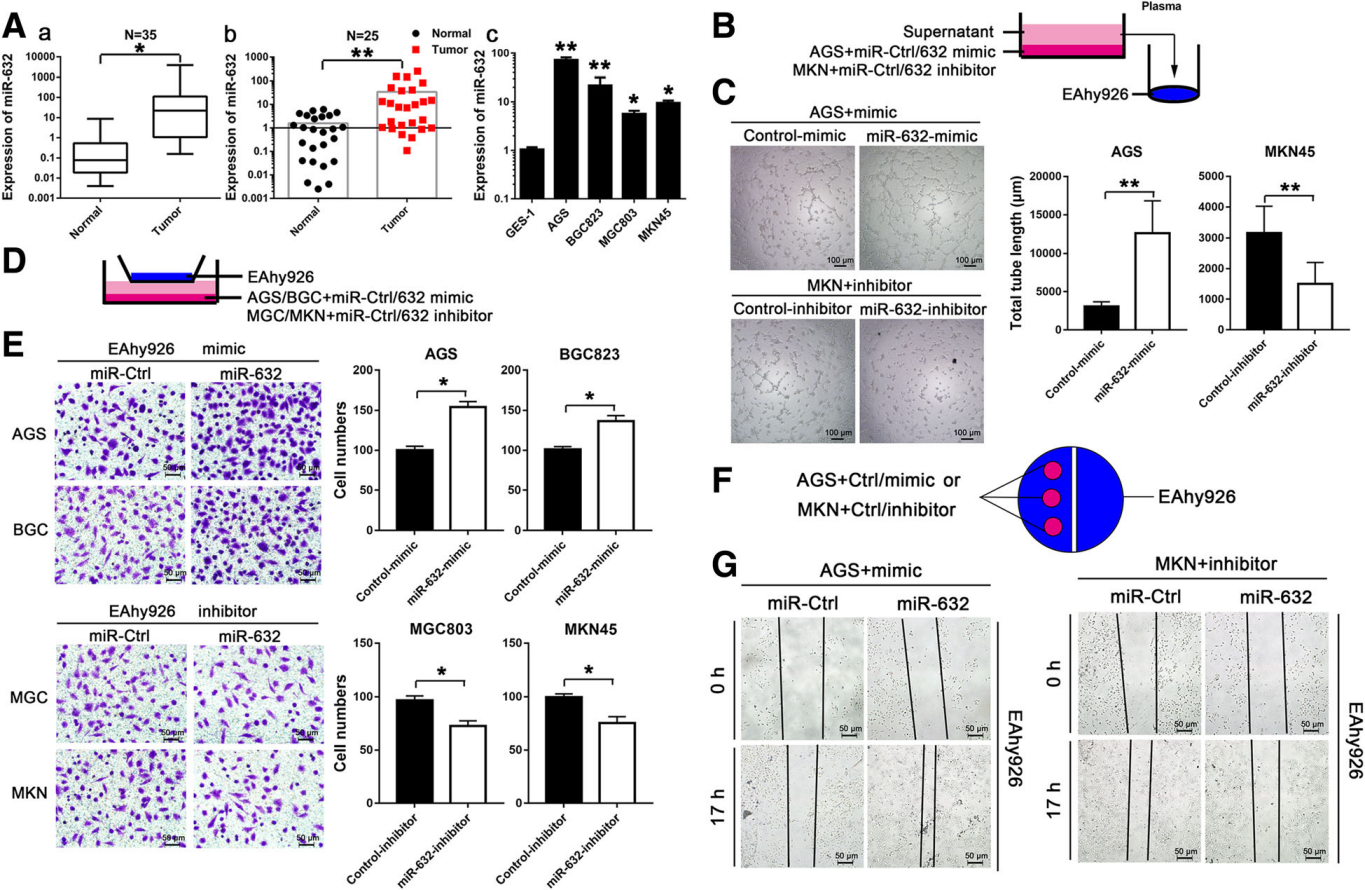
miR-632 is highly expressed in GC and improved GC cell angio-tube formation and endothelial cell recruitment. **a** The expression of miR-632 in GC tissues (a, *n* = 35) and serum (b, *n* = 25) from both the normal population and GC patients. (c) The expression of miR-632 was significantly higher in GC cells. **b** Schematic diagram showing the miR-632-mediated tube formation assay in GC cells. **c** Tube formation was accelerated by miR-632-mimic in AGS cells (upper left panels). miR-632-inhibitor decreased tube formation compared with the control in MKN45 cells (lower left panels). The histograms present the total tube length (mean ± SD) from three random fields at high magnification (right panels). **d** Schematic diagram showing the miR-632-mediated co-culture system using for endothelial cell Transwell assays in GC cells. **e** miR-632 mimic increased endothelial cell recruitment in AGS and BGC823 cells (upper left panels). The histograms present the cell numbers (mean ± SD) from three random fields at high magnification (upper right panels). miR-632-inhibitor suppressed endothelial cell recruitment in MGC803 and MKN45 cells (lower left panels). The histograms present the cell numbers (mean ± SD) from three random fields at high magnification (lower right panels). **f** Schematic diagram showing miR-632-mediated endothelial cell recruitment in wound healing assay of GC cells. **g** Wound healing was accelerated by miR-632-mimic in AGS cells (left panels). miR-632-inhibitor decelerated healing after scratching compared with the control in MKN45 cells (right panels). The experiments were performed at least three times independently. * *P* < 0.05; ** *P* < 0.01

We measured the expression of miR-632 in both GC tissues and serum. We found that the expression of miR-632 was upregulated in GC tissues (Fig. 1a, left panel, *n* = 35, *P* < 0.05) and in serum of GC patients (Fig. 1a, middle panel, *n* = 25, *P* < 0.01) compared with the corresponding controls. We also analysed the expression of miR-632 in different cell lines. Figure 1c shows that the expression of miR-632 was upregulated in GC cells compared with normal gastric epithelial cells.

### miR-632 improves tube formation and endothelial cell recruitment in GC cells

We transfected AGS and BGC823 cells with chemically synthesized miR-632-mimic oligonucleotides (25 nM) or a corresponding control (Additional file 1: Figure S1A, *P* < 0.01) and transfected MGC803 and MKN45 cells with miR-632-inhibitor oligonucleotides (50 nM) or a corresponding control (Additional file 1: Figure S1B, *P* < 0.01). After 24 h, the supernatants were collected and used to incubate EAhy926 endothelial cells. Then, angiogenesis tube formation assays were performed (Fig. 1b). Figure 1c shows that miR-632-mimic increased angiogenesis tube formation in AGS cells and miR-632-inhibitor suppressed angiogenesis tube formation in MKN45 cells compared with the corresponding controls (Fig. 1c, *P* < 0.01).

We generated a GC cell co-culture system to perform endothelial cell recruitment assays. GC cells were treated with miR-632-mimic or inhibitor or their corresponding controls in microplates to recruit endothelial EAhy926 cells placed in the chambers (Fig. 1d). Figure 1e shows that miR-632-mimic increased endothelial cell recruitment in AGS and BGC cells (upper panels) and miR-632-inhibitor suppressed endothelial cell recruitment in MGC803 and MKN45 cells (lower panels) compared with the corresponding controls (*P* < 0.05). GC cells were transfected with miR-632-mimic or inhibitor on microslides to recruit EAhy926 cells (Fig. 1f). Figure 1g shows that miR-632-mimic accelerated healing of a cell scratch wound compared with the control (left panels), while miR-632-inhibitor decelerated scratch wound healing compared with the control in MKN45 cells (right panels).

### miR-632 expression is negatively associated with TFF1 in GC

**Fig. 2:**
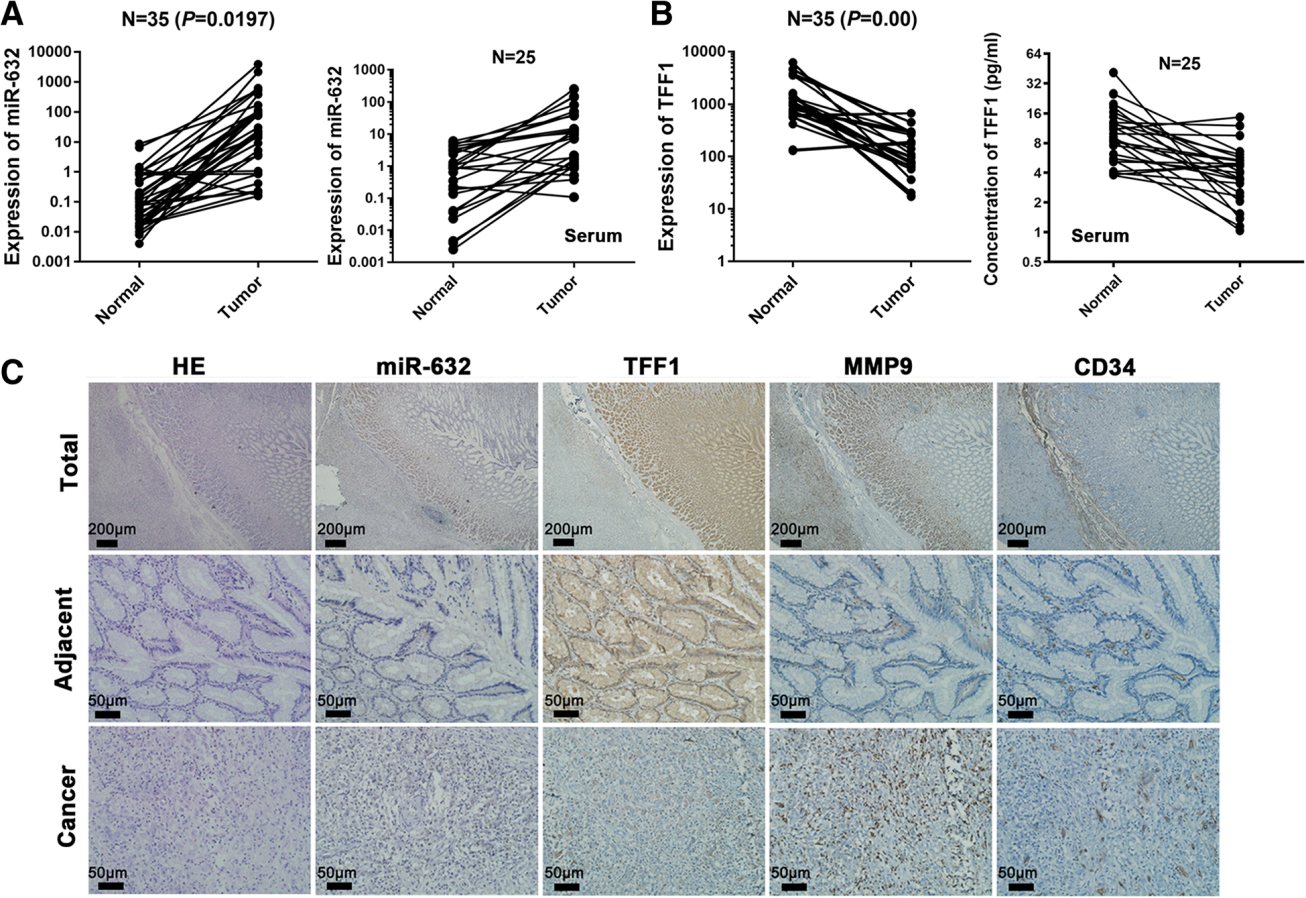
The expression of miR-632 was negatively associated with TFF1 in GC. **a** The expression of miR-632 in GC tissues compared with adjacent non-cancerous tissues (left panel, *n* = 35) and in GC serum compared with healthy serum (right panel, *n* = 25) measured via realtime PCR. **b** The expression of TFF1 in GC tissues compared with that in adjacent non-cancerous tissues measured by realtime PCR (left panel, *n* = 35). The concentration of TFF1 was detected via ELISA in GC serum compared with healthy serum (right panel, *n* = 25). **c** miR-632 expression was detected via in situ hybridization, and TFF1, MMP9 and CD34 expression was examined through immunohistochemiscal staining

We collected tumour samples from 35 human GC patients and measured miR-632 expression in human GC tissues. miR-632 expression was significantly up-regulated in GC lesions compared with adjacent non-cancerous tissues (Fig. 2a, left panel, *n* = 35, *P* < 0.05), and was negatively related to low expression of TFF1 (Fig. 2b, left panel, *n* = 35, *P* < 0.01). We also found that miR-632 was highly expressed in GC serum compared with healthy serum (Fig. 2a, right panel, *n* = 25, *P* < 0.05), and was negatively related to low concentration of TFF1 (Fig. 2b, right panel, *n* = 25, *P* < 0.05). Additional samples were collected, and the expression of miR-632 was measured by performing an in situ hybridization assay in 42 pairs of GC and adjacent non-cancerous tissues (Fig. 2c). The positive expression rate of miR-632 in GC was 71.43%, significantly higher than that in adjacent non-cancerous mucosa (26.19%) (Table 1, *P* < 0.00). The clinical characteristics related to miR-632 expression are analysed in Table 2. We also detected the expression of TFF1 and the angiogenesis-related biomarkers MMP9 and CD34 in an immunohistochemistry staining assay (Fig. 2c). We found that TFF1 exhibited lower expression while MMP9 and CD34 showed higher expression in tumour tissues compared than in adjacent non-cancerous tissues. Thus, miR-632 expression is negatively associated with TFF1 expression in GC cells.

**Table 1:** miR-632 expression in 42 pairs of GC tissues. The positive expression rate of miR-632 in 42 GC tissues pairs with normal gastric mucosa (*P* = 0.00)

Group	miR-632		Positive rate (%)	*P* value
	Positive	Negative		
Normal gastric mucosa	11	31	26.19	0.000
Gastric cancer	30	12	71.43

**Table 2:** Clinical characteristics related to miR-632

Characteristics	miR-632		Positive rate (%)	*P* value
	Positive	Negative		
Gender				
Male	22	5	81.48	0.114
Female	8	7	53.33	
Age (yr)				
60 yr. or younger	13	6	68.42	0.695
Over 60 yr	17	6	73.91	
Differentiation				
Moderate	21	8	72.41	1.000
Poor	9	4	69.23	
Stage				
I + II	27	7	79.41	0.054
III + IV	3	5	37.50	
Lymph nodes involvement				
Yes	3	3	50.00	0.443
No	27	9	75.00	

### miR-632 negatively regulates TFF1 in GC cells

**Fig. 3:**
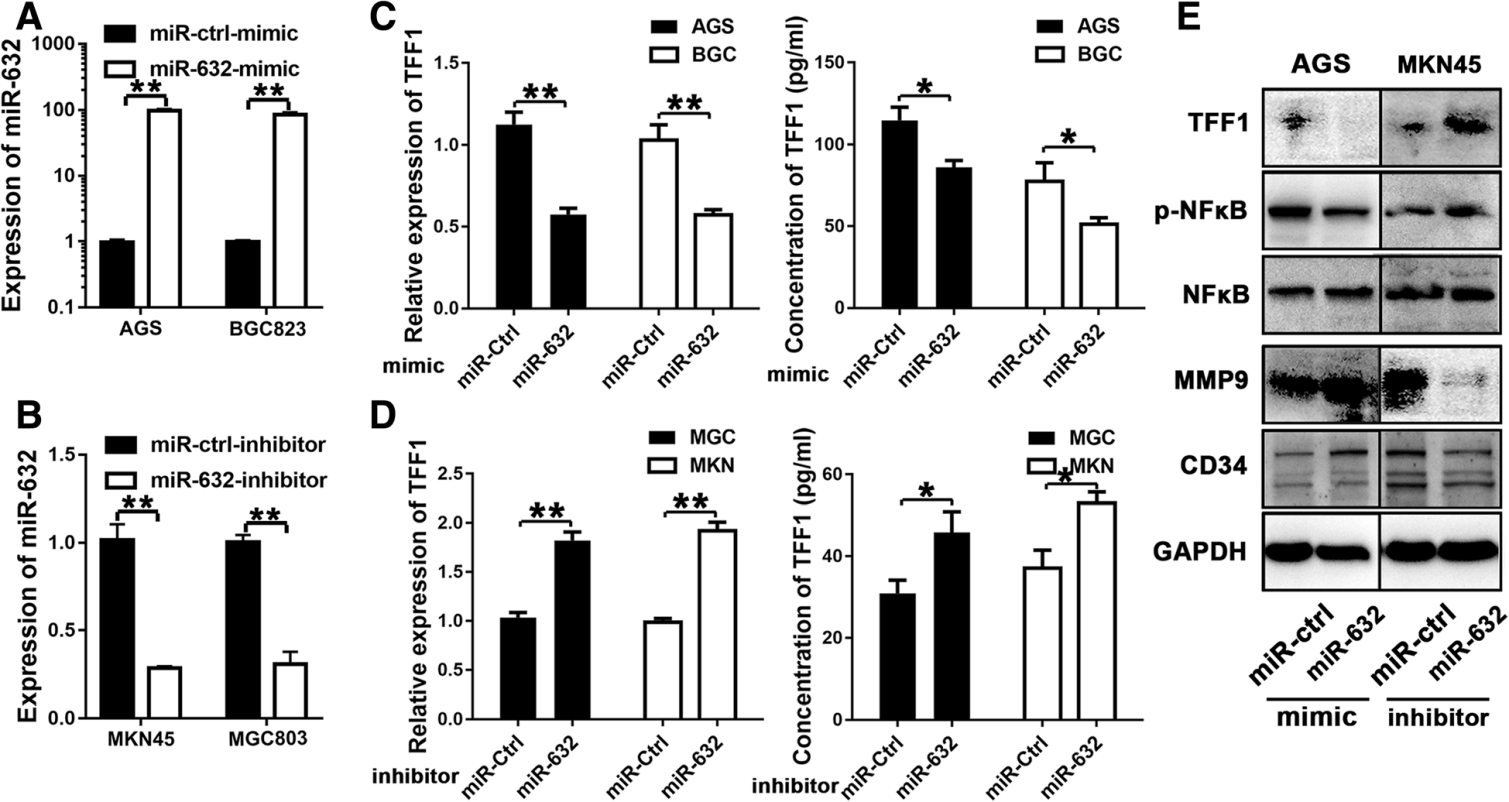
miR-632 negatively regulates TFF1 expression in GC cells. **a** miRNA mimic upregulated miR-632 expression compared with the negative control in AGS and BGC823 cells. **b** miRNA inhibitor downregulated miR-632 expression compared with the negative control in MKN45 and MGC803 cells. **c** miR-632-mimic reduced TFF1 expression (left panel) and secretion (right panel) in AGS and BGC823 cells compared with the negative control. **d** miR-632-inhibitor increased TFF1 expression (left panel) and secretion (right panel) compared with the negative control in MGC803 and MKN45 cells. **e** Western blot analysis of miR-632-mimic or inhibitor treatment in GC cells. The experiments were performed at least three times independently. *P < 0.05; **P < 0.01

We transfected AGS and BGC823 cells with 25 nM miR-632-mimic (Fig. 3a, *P* < 0.01) and found that miR-632 reduced TFF1 expression by at least 50% compared with the negative control (Fig. 3c, left panel, *P* < 0.01). Next, we detected the concentration of TFF1 secreted by GC cells and found that miR-632-mimic decreased TFF1 secretion in AGS and BGC823 cells (Fig. 3c, right panel, *P* < 0.05). In addition, we treated MGC803 and MKN45 cells with 50 nM miR-632-inhibitor (Fig. 3b, *P* < 0.01) and found that TFF1 expression was 1.75-fold higher than in the negative control cells (Fig. 3d, left panel, *P* < 0.01). In addition, miR-632-inhibitor increased TFF1 secretion in MGC803 and MKN45 cells (Fig. 3d, right panel, *P* < 0.05). Western blotting was performed (Fig. 3e) to verify the expression of related biomarkers in GC cells. We found that miR-632-mimic reduced the expression of TFF1 at the protein level in AGS cells compared with the corresponding control cells (Fig. 3e, left panels). However, NFκB phosphorylation showed no significant changes. In addition, we measured angiogenesis-related biomarkers and found that miR-632-mimic upregulated MMP9 and CD34 expression in tumour tissues (Fig. 3e, left panels). Moreover, miR-632-inhibitor increased the expression of TFF1 in MKN45 cells and downregulated the expression of MMP9 and CD34 (Fig. 3e, right panels).

### TFF1 reverses angiogenesis mediated by miR-632 in GC cells

**Fig. 4:**
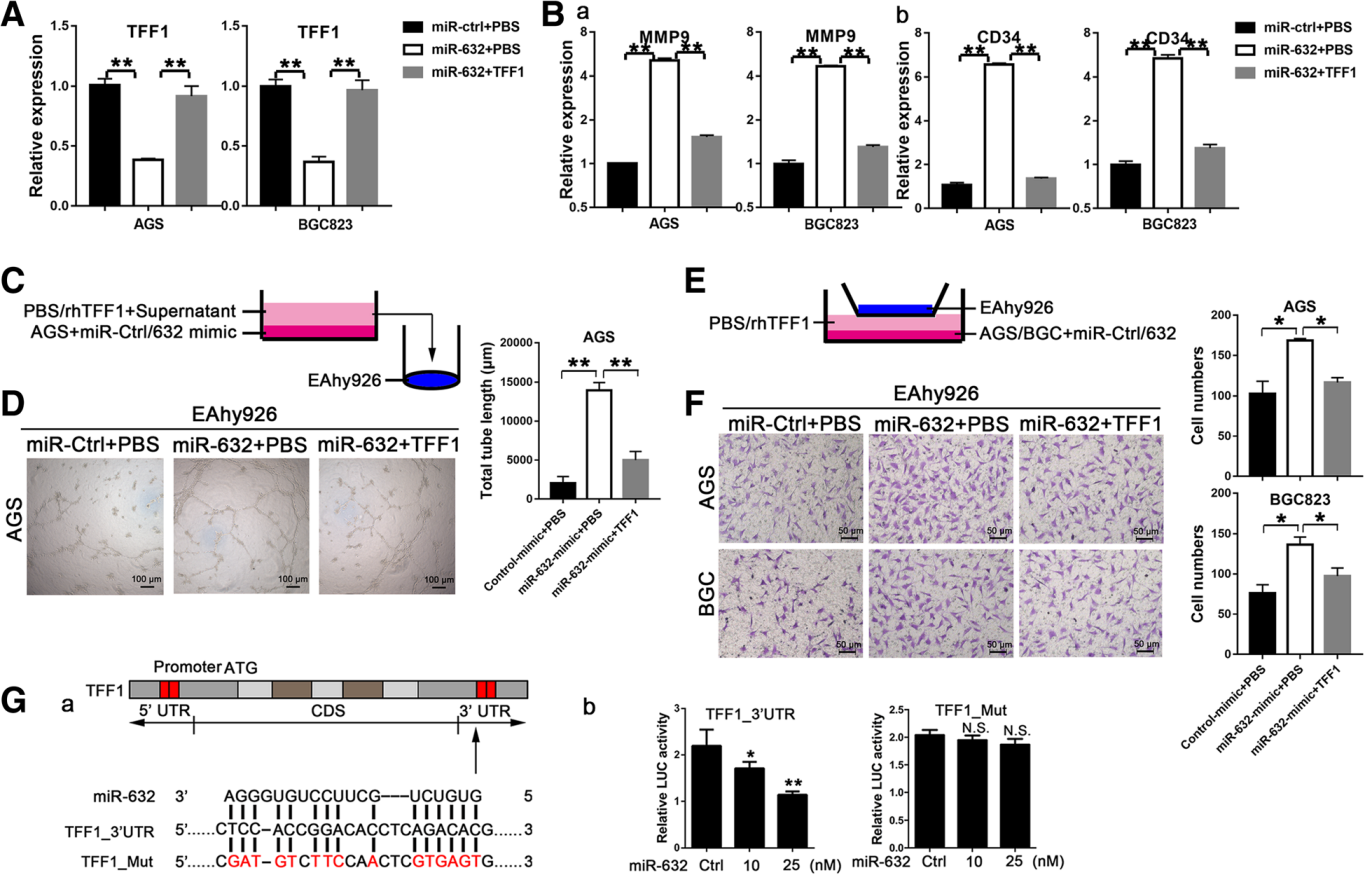
TFF1 is a target gene of miR-632. **a** Recombinant TFF1 protein rescued TFF1 expression inhibited by miR-632-mimic in AGS and BGC823 cells. **B** The expression of MMP9 (a) and CD34 (b) with recombinant TFF1 treatment in miR-632-mimic-transfected AGS and BGC cells. **c** Schematic diagram showing the miR-632-mediated co-culture system for angio-tube formation assays with or without recombinant TFF1 in GC cells. **d** Recombinant TFF1 reversed tube formation mediated by miR-632 (left panels). The histograms present the total tube length (mean ± SD) from three random fields at high magnification (right panel). **e** Schematic diagram showing the miR-632-mediated co-culture system used for endothelial cell Transwell assays with or without TFF1 recombinant protein in GC cells. **f** TFF1 recombinant protein reversed endothelial cell recruitment mediated by miR-632 (left panels). The histograms present the cell numbers (mean ± SD) from three random fields at high magnification (right panels). **G** Schematic diagram showing miR-632 and potential binding regions in the 3’UTR of TFF1 (a). (b) Relative luciferase activity of the TFF1–3’UTR reporter (left panel) and mutated-3’UTR reporter (right panel) in cells treated with miR-632-mimic compared with the control. The experiments were performed at least three times independently. *P < 0.05; **P < 0.01

Recombinant TFF1 protein (1 μg/mL) was used to rescue the TFF1 downregulation mediated by miR-632 in AGS and BGC823 cells (Fig. 4a, *P* < 0.01). After recombinant TFF1 treatment, the MMP9 (Fig. 4B-a, *P* < 0.01) and CD34 (Fig. 4B-b, *P* < 0.01) upregulation mediated by miR-632 was significantly decreased. To confirm the effect of TFF1 on angiogenesis mediated by miR-632, angio-tube formation (Fig. 4c) and endothelial cells recruitment (Fig. 4e) assays were performed after recombinant TFF1 treatment in AGS and BGC823 cells. Recombinant TFF1 reversed the tube formation increased by miR-632-mimic in AGS cells (Fig. 4d, *P* < 0.01), and suppressed the endothelial cell recruitment accelerated by miR-632-mimic in AGS and BCG823 cells (Fig. 4e and f, *P* < 0.05). Thus, miR-632 improves angiogenesis in a TFF1-dependent manner in GC cells.

### TFF1 is a miR-632 target gene

We generated dual-luciferase reporter plasmids containing the full-length 3’UTR of TFF1 (pmirGLO-TFF1) or mutated potential binding sites (pmirGLO-Mut) to confirm whether miR-632 regulated TFF1 directly (Fig. 4G-a). Compared with the control, the relative luciferase activity of the pmirGLO-TFF1 reporter was markedly suppressed, with 83% expression after treatment with 10 nM mimic and 51% expression after treatment with 25 nM mimic (Fig. 4G-a, right panel, *P* < 0.05). However, the activity of the reporter containing a mutated site exhibited no significant alterations in cells transfected with miR-632-mimic (Fig. 4G-b). Therefore, we concluded that TFF1 is a target gene of miR-632 and that miRNA-632 negatively regulates TFF1 expression by binding to its 3’UTR.

Thus, we conclude that miR-632 promotes GC progression by accelerating angiogenesis in a TFF1-dependent manner.

## Discussion

In our current study, we demonstrated that miR-632 promotes GC progression by accelerating angiogenesis in a TFF1-dependent manner. Our results showed that miR-632 is highly expressed in GC tissue and serum and negatively associated with TFF1 in GC. Here, we detected miR-632 in serum to provide an experimental basis for future non-invasive rapid diagnosis of GC using peripheral blood samples. miR-632 improves tube formation and endothelial cell recruitment by negatively regulating TFF1 in GC cells. Recombinant TFF1 reversed angiogenesis mediated by miR-632. TFF1 is a target gene of miR-632.

As important regulators of gene expression, miRNAs have not only been implicated in various signalling pathways but also in embryonic development, tissue homeostasis, stem cell transition, anticancer therapy, and other biological processes [29–31]. A miRNA-associated diagnosis provides predictive power to inform early GC patients and has the potential to be applied in endoscopic treatment [14]. We previously found that miR-218-5p and miR-423-5p regulate GC proliferation and invasion, respectively [27, 28]. miR-218-5p regulates GC cell the proliferation by targeting TFF1 in an Erk1/2-dependent manner. miR-423-5p regulates cell proliferation and invasion by targeting TFF1 in GC cells. We previously suggested that TFF1 improves gastric mucosal protection and epithelial integrity [25]. Trefoil peptides are also used to protect against mucosal injury via oral administration or other approaches [32, 33]. Additionally, increased TFF1 expression in para-carcinoma tissue suggests that TFF1 is associated with tumour suppression and differentiation. TFF1 is involved in inhibition of tumour invasion and migration, and it may be used as a target to enhance the chemotherapy sensitivity by regulating apoptosis resistance [34]. In addition, miRNAs can regulate TFF1 expression and secretion. In our present study, we found that miR-632 may inhibit TFF1 expression and secretion in GC cells.

## Conclusions

Therefore, our research demonstrated that miR-632 is upregulated in GC tissue and serum and negatively associated with TFF1. miR-632 improves tube formation and endothelial cell recruitment by negatively regulating TFF1 in GC cells. We conclude that miR-632 promotes GC progression by accelerating angiogenesis in a TFF1-dependent manner.

## Additional file

Additional file 1: **Figure S1**. Human GC cells were transfected with miR-632-mimic or inhibitor. (A) miRNA mimic upregulated miR-632 expression compared with the negative control in AGS and BGC823 cells. (B) miRNA inhibitor downregulated miR-632 expression compared with the negative control in MKN45 and MGC803 cells. The experiments were performed at least three times independently. **P < 0.01. (DOCX 233 kb)

## Abbreviations

3′-UTR: 3′-untranslated region
GC: gastric cancer
miRNA: miR - microRNA
MMP: matrix metalloprotein
TFF1: trefoil factor 1
