# Supplementary figures and images for: miR-632 promotes gastric cancer progression by accelerating angiogenesis in a TFF1-dependent manner
Source: BMC Cancer. 2019 Jan 7;19:14. doi: 10.1186/s12885-018-5247-z (PMC6322242; doi:10.1186/s12885-018-5247-z)

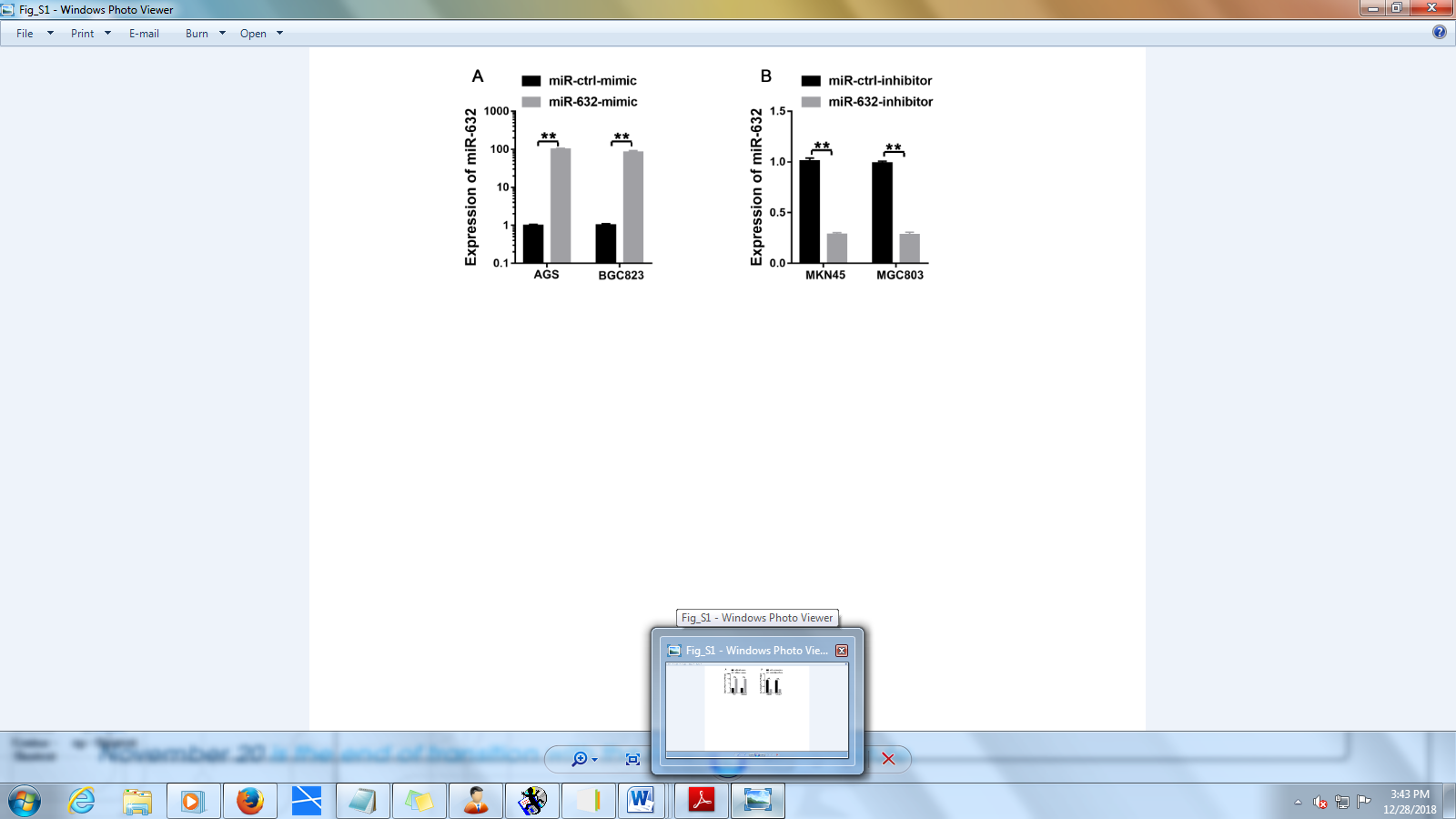

Supplement: Supplementary file 1 — Figure S1. Human GC cells were transfected with miR-632-mimic or inhibitor. (A) miRNA mimic upregulated miR-632 expression compared with the negative control in AGS and BGC823 cells. (B) miRNA inhibitor downregulated miR-632 expression compared with the negative control in MKN45 and MGC803 cells. The experiments were performed at least three times independently. **P < 0.01. (DOCX 233 kb) [file 12885_2018_5247_MOESM1_ESM.docx]
